# Bipolarization of Risk Perception about the Health Effects of Radiation in Residents after the Accident at Fukushima Nuclear Power Plant

**DOI:** 10.1371/journal.pone.0129227

**Published:** 2015-06-09

**Authors:** Makiko Orita, Naomi Hayashida, Yumi Nakayama, Tetsuko Shinkawa, Hideko Urata, Yoshiko Fukushima, Yuuko Endo, Shunichi Yamashita, Noboru Takamura

**Affiliations:** 1 Department of Global Health, Medicine, and Welfare, Atomic Bomb Disease Institute, Nagasaki University Graduate School of Biomedical Sciences, Nagasaki 8528523, Japan; 2 Department of Nursing, Nagasaki University Graduate School of Biomedical Sciences, Nagasaki 8528523, Japan; 3 Division of Strategic Collaborative Research, Center for Promotion of Collaborative Research on Radiation and Environment Health Effects, Atomic Bomb Disease Institute, Nagasaki University Graduate School of Biomedical Sciences, Nagasaki 8528523, Japan; 4 Department of Radiation Medical Sciences, Atomic Bomb Disease Institute, Nagasaki University Graduate School of Biomedical Sciences, Nagasaki 8528523, Japan; 5 Hirosaki University School of Health Sciences, Hirosaki 0368560, Japan; 6 Kawauchi Municipal Government, Fukushima 9791201, Japan; Northwestern University Feinberg School of Medicine, UNITED STATES

## Abstract

The late health effects of low-dose rate radiation exposure are still a serious public concern in the Fukushima area even four years after the accident at Fukushima Daiichi Nuclear Power Plant (FNPP). To clarify the factors associated with residents’ risk perception of radiation exposure and consequent health effects, we conducted a survey among residents of Kawauchi village in May and June 2014, which is located within 30 km of FNPP. 85 of 285 residents (29.8%) answered that acute radiation syndrome might develop in residents after the accident, 154 (54.0%) residents responded that they had anxieties about the health effects of radiation on children, and 140 (49.1%) residents indicated that they had anxieties about the health effects of radiation on offspring. Furthermore, 107 (37.5%) residents answered that they had concerns about health effects that would appear in the general population simply by living in an environment with a 0.23 μSv per hour ambient dose for one year, 149 (52.2%) residents reported that they were reluctant to eat locally produced foods, and 164 (57.5%) residents believed that adverse health effects would occur in the general population by eating 100 Bq per kg of mushrooms every day for one year. The present study shows that a marked bipolarization of the risk perception about the health effects of radiation among residents could have a major impact on social well-being after the accident at FNPP.

## Introduction

On 11 March 2011, a magnitude 9.0 earthquake struck Japan, followed within the hour by the first of a series of tsunamis that hit the coast of the Tohoku region of northern Japan. The natural disaster caused immense damage to infrastructure, the economy, and the very social fabric itself [1]. It also led to severe damage to the Fukushima Daiichi Nuclear Power Plant (FNPP), including core meltdown in the three reactors and the release of large amounts of radionuclides into the air.

In response to the accident, the Japanese and Fukushima prefectural governments issued instructions for the evacuation of settlements within a 20-km radius of FNPP just after the accident. Furthermore, beyond that inner circle, certain areas where concerns remained that cumulative doses of radiation might reach 20 mSv per year, were designated Deliberate Evacuation Areas. As a result, almost 110,000 local residents evacuated their homes; many residents voluntarily evacuated outside of Fukushima prefecture entirely [2–5]. Monitoring of food and drinking water by Japanese and prefectural governments began on 16 March 2011. Selected foodstuffs (milk, vegetables, grains, meat, fish, etc.) containing radioactive material that exceeded the provisional regulation values as recommended on 17 March 2011 by Japan’s Ministry of Health, Labour and Welfare were prohibited from distribution on 21 March 2011 and from consumption on 23 March 2011 [1, 4, 5]. In spite of these and other efforts to minimize external and internal radiation exposure doses, 47,149 residents of Fukushima Prefecture have remained evacuated to other prefectures as of August 2014 [6].

Since the accident, measurements of external and internal radiation exposure of residents surrounding the FNPP have been reported by several research institutions; they all suggest that external and internal radiation doses caused by the accident were relatively low and far from any direct health consequence in the general population [7–10]. Nevertheless, the health effects of radiation exposure remain a serious public concern in Fukushima. In May 2014, a popular Japanese cartoon stirred up residents’ anxiety by linking Fukushima to nosebleed, one of the typical manifestations of acute radiation syndrome (ARS) [11].

ARS is an acute illness caused by irradiation of the entire body by a high dose of radiation in a very short period of time (usually a matter of minutes). The typical syndrome of ARS is neurovascular syndrome, gastrointestinal syndrome, haematopoietic syndrome and cutaneous syndrome. During the prodromal period, loss of appetite, nausea, vomiting and diarrhea can occur. However, all of these symptoms usually disappear in a day or two when latent period follows. A period of illness follows can be characterized by predisposition to infection and bleeding related to falling blood counts. Death or a period of recovery follows the period of overt illness. The severity and time sequence of these phases depends on the dose and dose rate, usually ARS can be detected after an acute radiation dose as low as 0.5–1.0 Gy [12]. In the cartoon, the main character, a journalist, notes that the nosebleed that he experienced was caused by radiation exposure during a trip to FNPP. Such misleading information may well affect the public risk perception about the health effects of radiation in Fukushima.

From this point of view, it is essential to evaluate the risk perception of the health effects of radiation in residents and to implement a comprehensive risk communication strategy. In this study, we conducted a survey of residents of the frontline village of Kawauchi, which is located less than 30 km from FNPP, to clarify the factors associated with residents’ risk perception of radiation exposure and the consequent health effects.

## Materials and Methods

### Study Participants

**Fig 1:**
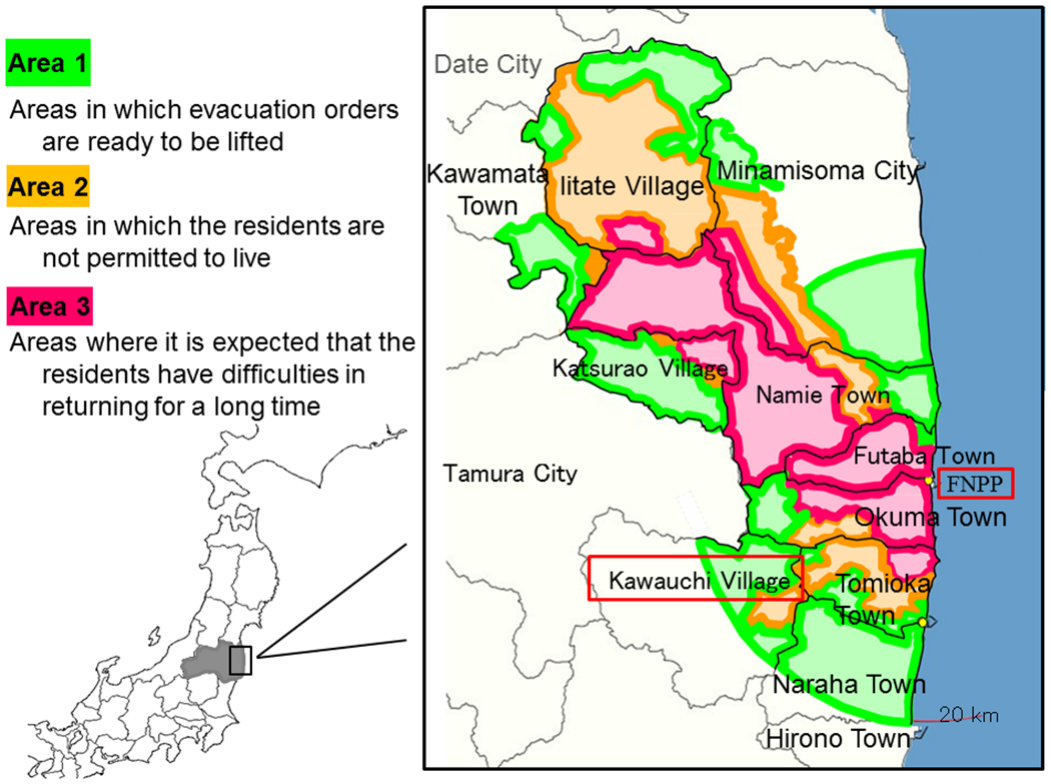
Location of Kawauchi in Fukushima.

The study was conducted in the village of Kawauchi in Fukushima prefecture in May and June 2014. Kawauchi is located less than 30 km from FNPP and was partially included in the Evacuation Order Area established within a 20-km radius from the FNPP (Fig 1). Almost all residents evacuated the village in the accident’s initial phase. On 31 January 2012, the mayor of the village declared that residents who lived at least 20 km away from FNPP could return to their homes because the Japanese Prime Minister had declared that the FNPP reactors had achieved a state of “cold shutdown” in December 2011 [13]. Since April 2014, all residents who lived within a 20-km radius from the FNPP are permitted to temporarily return to their houses, and in October 2014, the village decided to lift the evacuation order for the area 20 km or less from the FNPP. However, the number of residents who have actually returned to the village is still low. As of January 2015 only 1,581 of 2,739 (57.7%) residents have returned to Kawauchi, with the other residents still living in other cities [13, 14].

We initially distributed questionnaires to the almost 2,500 residents who were 18 years of age or older and lived in the village at the time of the accident. We obtained responses from 332 residents, and after excluding 47 residents for insufficient responses, we included 285 residents (141 men and 144 women) in the analysis. The study was approved by the ethics committee of the Nagasaki University Graduate School of Biomedical Sciences (project registration number 14031395). Before the study, we obtained permission from the village’s municipal government to implement the study.

### Questionnaire

The questionnaire for this study was developed based on our previous study [15], the mental health and lifestyle survey within the framework of the Fukushima Health Survey [16], and a Q & A that we published for residents of Fukushima Prefecture after the accident [17]. We asked about demographic variables including sex, age at the time of the study, employment status, residential location in the village at the time of the accident, returning to the village or not, living apart from family after the accident, and currently growing rice or vegetables. We included questions to evaluate the risk perception of the health effects of radiation in the survey, such as the health effects of radiation in children and on offspring and knowledge of the health effects of radiation of living in an environment with 0.23μSv per hour of ambient dose rate for one year (equivalent to 1 mSv per year) and of eating 100 Bq per kg (the current standard regulatory value of foods in Japan. The permissible radiation for mushrooms in Wuropen Union (EU) and USA is 500 Bq/kg and 1200 Bq/kg, respectively) of mushrooms every day for one year (equivalent to 0.05 mSv per year). We also included questions about whether residents thought that ARS might occur in residents due to the FNPP accident, whether they were reluctant to eat rice or vegetables produced in the village, and whether they were reluctant to undergo radiological examinations at a hospital. All questions were evaluated on a four point scale (1 = yes, 2 = probably yes, 3 = probably no, and 4 = no).

### Statistical Analysis

Answers were divided into two categories; “yes” and “probably yes” as “yes” and “probably no” or “no” as no. We divided age into two categories; ≤60 y and ≥61 y. Residential location was divided into two areas according to geographical location within the village. We identified the factors associated with risk perception for the health effects of radiation using the chi-square test. We also conducted logistic regression analysis and calculated odds ratios (OR) to identify the risk perception of the possible occurrence of ARS after the accident. P-values less than 0.05 were considered significant.

## Results

**Fig 2:**
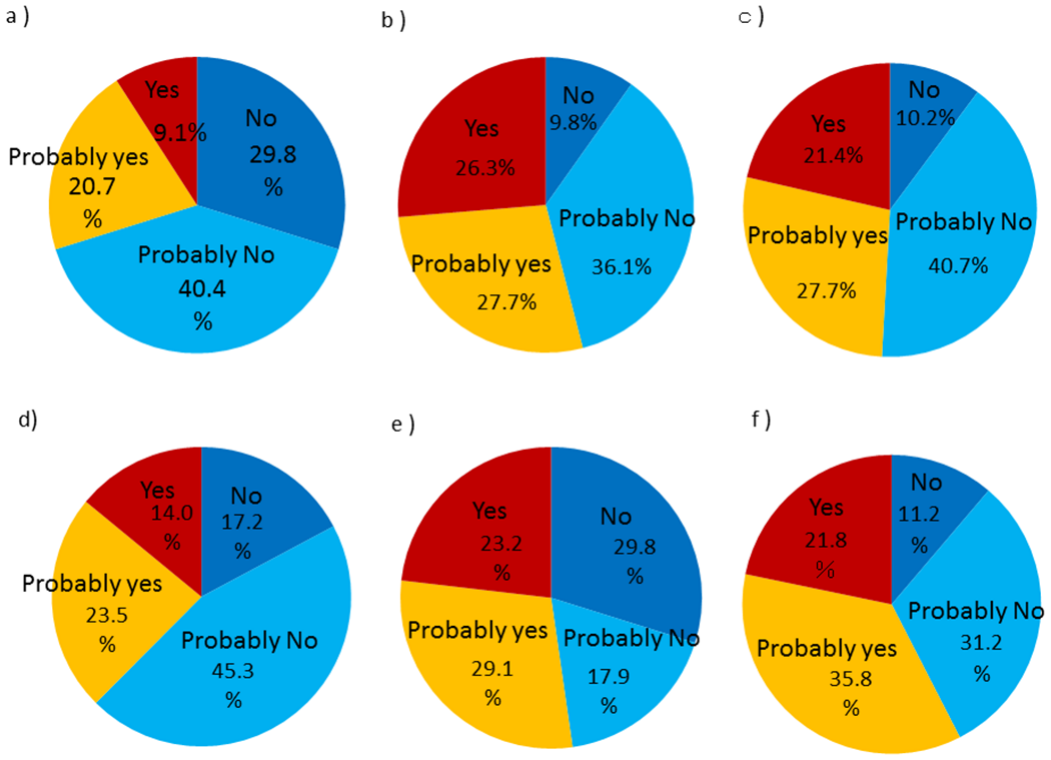
**Residents’ risk perception of the health effects of radiation:** a) “Do you think that that acute radiation syndrome might develop in residents due to radiation exposure following the Fukushima accident?” b) “Do you have anxiety about the health effects of radiation on children?” c) “Do you have anxiety about the health effects of radiation on offspring?” d) “Do you have anxiety that health effects would develop in the general population simply by living in an environment with a 0.23 μSv per hour ambient dose for one year? e) “Are you reluctant to eat rice or vegetables produced in the village?” f) “Do you believe that adverse health effects would occur in the general population by eating 100 Bq per kg of mushrooms for one year?”

The average age among study participants (N = 285) was not significantly different between men and women (65.2 ± 16.0 years vs. 64.8 ± 16.6 years, p = 0.86). 171 of the 285 residents (60.0%) have already returned to the village. 85 residents (29.8%) answered that the ARS might develop in residents after the accident at FNPP (Fig 2a), 154 (54.0%) residents answered that they had anxieties about the health effects of radiation on children (Fig 2b), and 140 (49.1%) residents reported that they had anxieties about the health effects of radiation on offspring (Fig 2c).

Fig 2d, 2e and 2f show the residents’ anxieties about the health effects of external and internal radiation exposures. 107 (37.5%) residents answered that they were concerned about health effects that would appear in the general population simply by living in an environment with a 0.23 μSv per hour ambient dose for one year (equivalent to 1 mSv per year) (Fig 2d), 149 (52.2%) residents answered that they were reluctant to eat rice or vegetables produced in the village (Fig 2e), and 164 (57.5%) residents believed that adverse health effects would occur in the general population by eating 100 Bq per kg of mushrooms every day for one year. (equivalent to 0.05 mSv per year) (Fig 2f).

85 respondents (29.8%) answered that ARS had developed due to the accident at FNPP (ARS+) and 200 (70.1%) answered that ARS had not developed due to the accident (ARS-). A significantly lower ARS+ residents than ARS- residents had already returned to the village (48.2% vs. 65.0%, p = 0.012) (Table 1). The following percentages were significantly higher among ARS+ than ARS-: those who had anxiety about the health effects of radiation on children (98.8% vs. 35.0%, p<0.001) and on offspring (95.3% vs. 29.5%, p<0.001); those who had anxieties about health effects that would appear in the general population simply by living for one year in an environment with a 0.23 μSv per hour ambient dose (82.4% vs. 18.5%, p<0.001); those who were reluctant to eat locally produced foods like rice or vegetables (85.9% vs 38.0%, p<0.001); those who believed that adverse health effects would occur in the general population by eating 100 Bq per kg of mushrooms every day for one year (83.5% vs 46.5%, p<0.001); and those who were reluctant to undergo radiological examinations at a hospital (63.5% vs. 21.0%, p<0.001).

**Table 1:** Residents’ demographic factors by risk perception for acute radiation syndrome (ARS) might develop for general population by the FNPP accident.

Variable and Questions	ARS might occur. (n = 85, %)	ARS might not occur. (n = 200, %)	P-Value
Are you a male?	42 (49.4)	99 (50.6)	1.000
Are you 60 years of age or older?	66 (77.6)	140 (70.0)	0.197
Did you live in Kami-Kawauchi, not Shimo-Kawauchi before the accident?	20 (23.5)	106 (53.0)	<0.001
Did you return to the village?	41 (48.2)	130 (65.0)	0.012
Do you live apart from your family after the accident?	41 (48.2)	88 (44.0)	0.519
Do you currently work?	25 (29.4)	87 (43.5)	0.034
Do you currently make rice or vegetables?	23 (27.1)	97 (48.5)	0.001
Do you have anxiety about the health effects of radiation on children?	84 (98.8)	70 (35.0)	<0.001
Do you have anxiety about the health effects of radiation on fetal development?	81 (95.3)	59 (29.5)	<0.001
Do you have anxiety about health effects would appear in the general population simply by living in an environment with a 0.23 μSv per hour ambient dose for one year?	70 (82.4)	37 (18.5)	<0.001
Are you reluctant to eat rice or vegetables produced in the village?	73 (85.9)	76 (38.0)	<0.001
Do you believe that adverse health effects would occur in the general population by eating 100 Bq per kg of mushrooms for one year?	71 (83.5)	93 (46.5)	<0.001
Are you reluctant to radiological examination in the hospital?	54 (63.5)	42 (21.0)	<0.001

Note: Number refers to people within the ARS+ or ARS- group that responded with a yes. The percentages refer to the fraction of people within the ARS+ or ARS- group that responded with a yes.

Logistic regression analysis revealed that anxiety about the health effects of radiation in children (OR = 31.01, 95%CI: 3.35–286.65, p = 0.002) and on offspring (OR = 4.73, 95%CI: 1.25–17.90, p = 0.022), concerns about health effects that would appear in the general population simply by living in an environment with a 0.23 μSv per hour ambient dose for one year (OR = 6.87, 95%CI: 2.67–17.71, p<0.001), and worries about the health effects of radiation of eating 100 Bq per kg of mushrooms every day for one year (OR = 3.54, 95%CI: 1.13–11.12, p = 0.030) were independently associated with residents’ risk perception of ARS after the accident.

## Discussion

The present study shows that 29.8% of the residents answered that ARS had developed in residents after the accident, 54.0% of the residents answered that they had anxieties about the health effects of radiation on children, and 49.1% residents answered that they had anxieties about the health effects of radiation on offspring. Our study also showed that 37.5% of residents responded that they were worried about the health effects of radiation on the general population of living in an environment with 0.23 μSv per hour of ambient dose rate for one year (equivalent to 1 mSv of radiation per year), 52.2% indicated that they were reluctant to eat locally produced foods like rice or vegetables, and 57.5% were concerned about the health effects of radiation on the general population by eating 100 Bq per kg of mushrooms every day for one year (equivalent to 0.05 mSv per year). Furthermore, logistic regression analysis revealed that anxieties about the health effects of radiation in children and offspring, concerns about the health effects of radiation by living in the environment with 0.23 μSv per hour ambient dose rate for one year, and worries about the health effects of radiation by eating 100 Bq per kg of mushrooms every day for one year were strongly associated with residents’ risk perception of ARS after the accident. These results suggest a markedly bipolar nature of the risk perception of the health effects of radiation among residents after the FNPP accident.

A 2012 study conducted one year after the accident within the framework of a Fukushima Health Survey showed that among 39,495 subjects who resided in Fukushima Prefecture at time of the accident, 6,304 (19.3%) believed that ARS had developed from radiation exposure in Fukushima due to the accident, 12,840 (39.4%) believed that health effects such as malignancies would occur later in life, and 15,546 (48.0%) believed that negative genetic effects in offspring would occur due to radiation exposure in Fukushima [16]. During the more than three years since the accident, enormous efforts have been made by specialists to distribute accurate information through risk communication to the public. Nevertheless, our results show that, despite the passage of time, the risk perception of health effects remains the same in Fukushima.

ARS is caused by irradiation of the whole body or a focal site by a high dose of radiation over a very short period of time [12]. The best-known examples of ARS victims are the survivors of the 1945 Hiroshima and Nagasaki atomic bombs and the firefighters who responded first to the Chernobyl Nuclear Power Plant accident in 1986 [18]. Usually ARS can be detected after an acute radiation dose as low as 0.5–1.0 Gy [19]. On the other hand, dose estimates following the FNPP accident were at relatively low levels among the general population. In the Fukushima Medical Survey, the external radiation doses of residents who lived in the prefecture during the accident were estimated based on their behavior during the four months that followed [15, 20] and evaluated at less than 1 mSv in 62.0% of individuals, less than 2 mSv in 94.0%, less than 3 mSv in 99.4%, and less than 5 mSv in 99.8% [20]. These results showed that external radiation doses among residents in Fukushima were far below the levels that cause ARS.

While high-dose exposure in experimental animals can cause various disorders in offspring, no evidence of clinical or subclinical effects has yet been found in the offspring of atomic bomb survivors [21]. Although an increased incidence of hereditary effects is not expected to appear among the general population following the FNPP accident, ensuring safety for women of child-bearing age and younger generations by providing accurate information about the health effects of radiation and by enhancing a perinatal care system is absolutely essential.

In cases of an exposure dose of more than 100 mSv, the incidence of cancer and the death rate increase with exposure doses [12]. Based on such scientific evidence, The International Commission on Radiological Protection (ICRP) recommends that the public be exposed to no more than 1 mSv of radiation per year under normal conditions [22, 23]. Even during radiation emergencies like the Chernobyl and Fukushima accidents, the ICRP recommends that annual exposure to radiation be limited, as far as possible, to the range of 20 to 100 mSv per year. Moreover, after the accident itself was over, the ICRP recommended that the dose level to optimize protection from radiation for people living in contaminated areas should be in the lower range of 1 to 20 mSv per year. Permissive radioactivity levels of foods and water during and after emergencies are determined based on this recommendation. The calculation of the dose level must be determined using a careful balance of many related factors, including the contamination levels of affected areas, the sustainability of social, economic, and environmental life, and people’s individual health situations [23]. Most importantly for public confidence and thus social stability, the dose limit does not represent a bright line between “safe” and “not safe” [24]. Nevertheless, our results show that such concepts of radiation protection were not fully understood by many Fukushima residents. Furthermore, such serious misunderstandings of radiation and its health effects might lead to distress and anxieties from loss of livelihoods [25], which can have a major impact on mental and social well-being. Specialists must rise to the challenge of overcoming the gap between the documented risk perception of residents in Fukushima and the realities of radiation safety and danger through thoughtful, patient, and diligent communication with the public. Furthermore, FNPP accident revealed the insufficient number of specialist who can take responsibilities of radiation risk communication. Establishment of training system for such specialists will be important in worldwide, as well as in Japan.

The present study has several limitations. First, there is the participant bias; this study was conducted only in Kawauchi, which limits the generalizability of the findings. Second, we could not obtain sufficient information on potential confounding factors such as detailed lifestyle habits. Further studies are needed to clarify the factors associated with risk perception of radiation exposure and health effects among residents in the FNPP area.

In conclusion, our study shows that a marked bipolarization of the risk perception of the health effects of radiation in residents could have a major impact on long-term social well-being after the accident at FNPP. It is vital for specialists to pursue a risk communication strategy with the public that accepts the serious misunderstandings among many residents even while presenting scientific evidence.
